# Four New Lignans from *Kadsura Interior* and Their Bioactivity

**DOI:** 10.3390/molecules23061279

**Published:** 2018-05-26

**Authors:** Jiu-Shi Liu, Jin Zhang, Yao-Dong Qi, Xiao-Guang Jia, Ben-Gang Zhang, Hai-Tao Liu

**Affiliations:** 1 Key Laboratory of Bioactive Substances and Resources Utilization of Chinese Herbal Medicine, Ministry of Education, Institute of Medicinal Plant Development, Chinese Academy of Medical Sciences & Peking Union Medical College, Beijing 100193, China; liujiushi90@163.com (J.-S.L.); jzhang419@163.com (J.Z.); ydqi@implad.ac.cn (Y.-D.Q.); bgzhang@implad.ac.cn (B.-G.Z.); 2 Institute of Traditional Chinese Medicine, Xinjiang Medical University, Urumqi 830011, China; jxyxj2003@163.com

**Keywords:** *Kadsura interior*, lignan, kadsutherin E-H, anti-platelet aggregation

## Abstract

A phytochemical investigation of the stems of *Kadsura interior* has led to an isolation of four new lignans, named kadsutherin E–H (**1**–**4**), together with two known lignans (**5**–**6**). The structures of the four new compounds were established on the basis of comprehensive spectroscopic analyses. Compounds **1**–**6** exhibited inhibition against adenosine diphosphate (ADP) induced platelet aggregation. Among the isolated compounds, kadsutherin F (**2**) showed the strongest anti-platelet aggregation activity with an inhibition of 49.47%.

## 1. Introduction

The stems of *Kadsura interior* A. C. Smith, an indigenous plant to Southern China (Yunnan), which was recorded in the Chinese pharmacopoeia (2015 Edition) as ‘Dian-Ji-Xue-Teng’, have been used for the treatment of menstrual irregularities, blood deficiencies, and other feminine disorders [1]. Various lignans [2,3,4] and triterpenoids [5] were isolated from this plant in previous studies. Many of these compounds have exhibited various beneficial activities, such as anti-lipidperoxidative [6,7,8], antitumor [3], anti-HIV [2,9], and anti-platelet aggregation [10].

**Figure 1:**
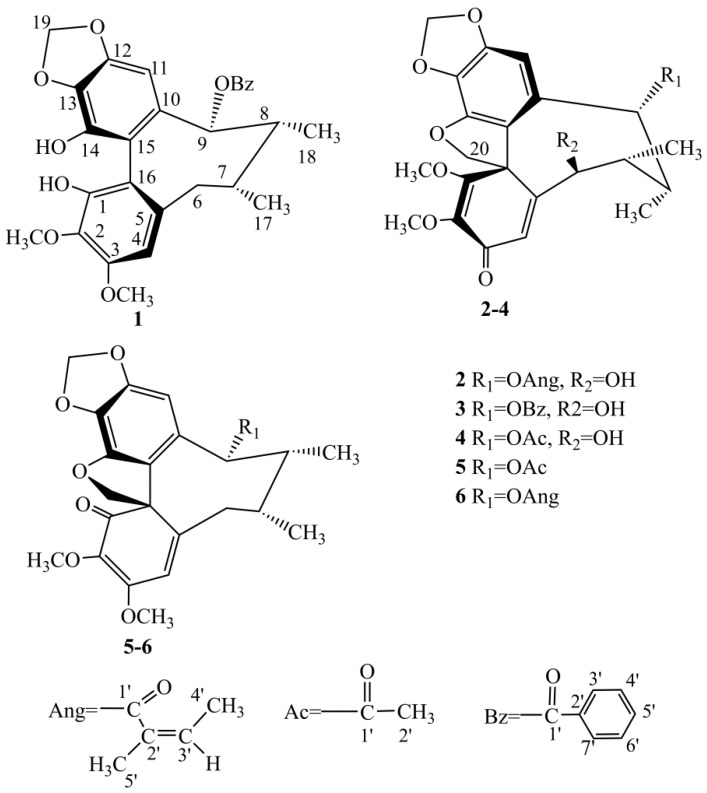
Structures of compounds **1**–**6.**

Our phytochemical investigation on *K. interior* led to the isolation and identification of the four new lignans, named as kadsutherin E-H (**1**–**4**), together with acetoxyl oxokadsurane (**5**) and heteroclitin D (**6**) (Figure 1). Compounds **1**–**6** exhibited the inhibition platelet aggregation induced adenosine diphosphate (ADP), with the range of 11.77–49.47%.

## 2. Results and Discussion

### 2.1. Four New Identified Compounds (**1**–**4**)

Kadsutherin E (**1**), which was obtained as a white powder, had the molecular formula C_28_H_28_O_8_, as it was revealed by its high-resolution electrospray ionization mass spectrometry (HRESIMS) (*m/z* 515.1691 [M + Na]^+^). The UV spectrum of **1** showed a maximum absorption at 221 nm, along with the ^1^H- and ^13^C-NMR spectra (Table 1), which indicated that **1** was a dibenzocyclooctene lignan [11,12].

**Figure 2:**
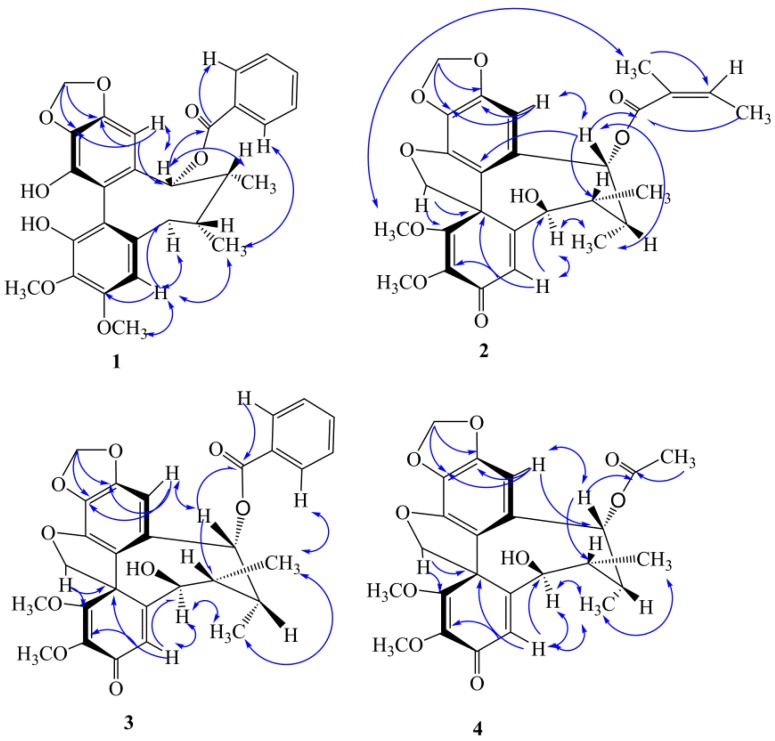
Key HMBC (H→C) correlations and selected NOESY (H ↔H) correlations of compounds **1**–**4**.

The ^1^H-NMR spectrum (Table 1) of **1** exhibited two aromatic singlets for a biphenyl moiety at *δ*_H_ 6.49 (H-11) and *δ*_H_ 6.60 (H-4), two singlets for methoxy groups at *δ*_H_ 3.40, 3.96 (3H each), one methylenedioxy (-OCH_2_O-) group at *δ*_H_ 5.97, 5.98 (1H each, d, *J* = 1.2 Hz), and three groups with characteristic signals of a benzoyl group at *δ*_H_ 7.48 (1H, dd, *J* = 7.2, 1.2, Hz, H-5′), 7.43 (2H, dd, *J* = 7.2, 1.2 Hz, H-3′, 7′) and 7.30 (2H, dd, *J* = 7.2, 7.2 Hz, H-4′, 6′). A cyclooctadiene ring was recognized from two secondary methyl doublets at *δ*_H_ 1.11 (CH_3_-17) and *δ*_H_ 1.15 (CH_3_-18), two methines at *δ*_H_ 2.20 (overlap, H-7, 8), an oxymethine at *δ*_H_ 5.92 (H-9), and a methylene at *δ*_H_ 2.72 and 2.77 (H_2_-6). The HMBC correlations of H-11 with C-12 (*δ*_C_ 147.9) and C-13 (*δ*_C_ 135.2), H-11 with C-9 (*δ*_C_ 83.5), two aromatic resonances (*δ*_H_ 5.97, 5.98) of the methylenedioxy moiety with C-12 and C-13, indicated that the methylenedioxy moiety was located at C-12 and C-13 (Figure 2). Two methylenedioxy group signals with C-3 (*δ*_C_ 151.2) and C-2 (*δ*_C_ 134.2) showed that two methylenedioxy groups were located at C-3 and C-2, respectively. The presence of a benzoyl group at C-9 was deduced from the HMBC correlation of H-9 with the *δ*_C_ 167.3 (C=O), 117.8 (C-10), 147.6 (C-15), 101.1 (C-11), 42.2 (C-8), and 34.8 (C-7).

The CD spectrum of **1** exhibited a positive Cotton effect around 214 nm and a negative Cotton effect around 242 nm, which suggested that **1** possessed an *S*-biphenyl conformation [13]. Compound **1** had a twist-boat-chair conformation because the correlated peaks of H-4 with CH_3_-17, H-9 with CH_3_-18, H-6a (*δ*_H_ 2.72, 1H, d, *J* = 13.8 Hz) with H-4, and CH_3_-17 with H-6a existed in the NOE spectrum (Figure 2). The absolute structure of kadsutherin E (**1**) was elucidated.

Kadsutherin F (**2**), which was obtained as a white powder, had the molecular formula C_28_H_28_O_8_, as revealed by its HRESIMS (*m*/z 521.1791 [M + Na]^+^). The UV spectrum of **2** showed a maximum absorption at 221 nm. The ^1^H-NMR and ^13^C-NMR spectra (Table 1 and Table 2) indicated that **2** was also a dibenzocyclooctene lignan. The characteristic proton signals at *δ*_H_ 4.46, 5.57(2H, d, *J* = 8.4 Hz) and a quaternary carbon signal at *δ*_C_ 83.7 indicated that **2** possessed a spiroenone ring, similar to kadsutherin D [14]. The ^1^H-NMR spectrum (Table 1) exhibited two aromatic singlets at *δ*_H_ 6.31 (H-4) and *δ*_H_ 6.39 (H-11), two methoxy groups at *δ*_H_ 3.69, 3.85 (3H each), one methylenedioxy (-OCH_2_O-) group at *δ*_H_ 5.99 (2H, s). In the cyclooctadiene ring, two doublet methyl groups at *δ*_H_ 1.02 and 1.04 (each 3H, d, *J* = 7.2 Hz) were located at C-7and C-8, respectively. Furthemore, the characteristic signals of an angeloxy group (*δ*_H_ 5.87, 1.77, 1.66 for H-3′, H-4′ and H-5′) were found in the ^1^H- and ^13^C-NMR spectra (Table 1 and Table 2). The HMBC correlations of H-11 with C-12 (*δ*_C_ 150.6) and C-13 (*δ*_C_ 129.8), H-11 with C-9 (*δ*_C_ 79.2), and two H-atoms (*δ*_H_ 5.97, 5.98) of the methylenedioxy moiety with C-12 and C-13, indicated that the methylenedioxy moiety was located at C-12 and C-13 (Figure 2). Moreover, two methoxy groups (1-OCH_3_, 2-OCH_3_) were deduced from the HMBC correlations of 1-OCH_3_ (*δ*_H_ 3.85, 3H, s) with C-1(*δ*_C_ 168.3), two characteristic proton signals (*δ*_H_ 4.46, 5.57, d, *J* = 8.4 Hz, CH_2_-20) with C-1, 2-OCH_3_ (*δ*_H_ 3.69, 3H, s) with C-2 (*δ*_C_ 134.8), H-4 with C-6 (*δ*_C_ 80.8), and H-4 with C-2. The presence of the angeloxy group at C-9 was deduced from the HMBC correlation of H-9 with the *δ*_C_ 167.3 (C=O), 130.1 (C-10), 120.4 (C-15), 100.2 (C-11), 42.3 (C-8), and 38.7 (C-7).

The CD spectrum of **2** exhibited a positive Cotton effect around 276 nm and a negative Cotton effect around 216 nm, which was contrary to the CD spectrum of the **1**, which suggested that **2** possessed an *R*-biphenyl conformation [14]. A twist-boat conformation of the cycloctadiene ring was deduced from the NOE correlations of H-9 with H-11, H-9 with CH_3_-18, H-6a (*δ*_H_ 4.13, 1H, d, *J* = 10.8 Hz) with H-4, and CH_3_-18 with H-4 (Figure 2). According to the above data, the structure of **2** was elucidated as kadsutherin F.

Kadsutherin G (**3**), which was obtained as a white powder, had the molecular formula C_29_H_28_O_9_, as it was revealed by its HRESIMS (*m*/*z* 543.1647 [M + Na]^+^). The ^1^H-NMR spectrum of **3** was similar to that of **2**, but the prominent difference in the ^1^H-NMR spectrum (Table 1) was the presence of a benzoyl group in **3**, which was substituted the angeloxy group in **2**. The data of the ^13^C-NMR spectrum of **3** (Table 2) also confirmed this deduction. In the HMBC spectrum of **3**, H-9 was correlated with the carbonyl carbon (*δ*_C_ 168.5, C=O) of the benzoyl group and *δ*_C_ 122.0 (C-10), 156.4 (C-15), 101.7 (C-11), 39.8 (C-8), and 44.0 (C-7), which clearly indicated that the benzoyl group was located at C-9.

The CD spectrum of **3** exhibited a positive Cotton effect around 311 nm and a negative Cotton effect around 224 nm, which suggested that **3** possessed an *R*-biphenyl conformation. A twist-boat conformation of **3** was deduced from the NOE correlations of CH_3_-17 with CH_3_-18, H-9b (*δ*_H_ 5.72, 1H, d, *J* = 7.2 Hz) with CH_3_-18, and H-6a (*δ*_H_ 4.26, 1H, d, *J* = 10.2 Hz) with CH_3_-17 (Figure 2). On the basis of the above data, the structure of **3** was educidated as kadsutherin G.

Kadsutherin H (**4**), which was obtained as a white powder, had the molecular formula C_24_H_26_O_9_, as it was revealed by its HRESIMS (*m*/*z* 481.1478 [M + Na]^+^). The ^1^H-NMR spectrum of **4** was also similar to that of **2**, but the prominent difference in the ^1^H-NMR spectrum (Table 1) was the presence of an acetoxy group in **4**, which was substituted the angeloxy group in **2**. The data of the ^13^C-NMR spectrum of **4** (Table 2) also confirmed this deduction. In the HMBC spectrum of **4**, H-9 was correlated with the carbonyl carbon (*δ*_C_ 171.9, C=O) of the acetoxy group and *δ*_C_ 122.2 (C-10), 146.6(C-15), 101.3 (C-11), 43.3 (C-8), and 40.4 (C-7), which clearly indicated that the acetoxy group was located at C-9.

The CD spectrum of **4** exhibited a positive Cotton effect around 245 nm and a negative Cotton effect around 223 nm, which suggested that **4** possessed an *R*-biphenyl conformation. Compound **4** had a twist-boat conformation, which was deduced from the NOE correlations of H-9 with H-11, H-9 with CH_3_-18, H-6a (*δ*_H_ 4.11, 1H, d, *J* = 10.2 Hz) with H-4, and CH_3_-18 with H-4 (Figure 2). Thus, **4** was elucidated as kadsutherin G.

A comparison of the NMR data with the reported values led to the identification of the structures of the known compounds **5**–**6** as acetoxyl oxokadsurane (**5**) [15] and heteroclitin D (**6**) [16].

### 2.2. Anti-Platelet Effects of Compounds 1–6

The anti-platelet effects of the compounds from the stems of the *K. interior* plants were tested in vitro, using the turbidimetric method in washed rat platelets that were induced by ADP (100 μM.). The anti-platelet aggregation data are shown in Table 3. The clinically applied anti-platelet agent aspirin, was used as the positive control. From the results of our anti-platelet aggregation tests, kadsutherin E (**1**), kadsutherin F (**2**), kadsutherin G (**3**), kadsutherin H (**4**), acetoxyl oxokadsurane (**5**), and heteroclitin D (**6**) exhibited inhibition (with inhibition in the range of 11.77–49.47%) against the ADP induced platelet aggregation. Among these compounds, kadsutherin F (**2**) showed the strongest anti-platelet aggregation activity with an inhibition of 49.47 ± 2.93%.

## 3. Materials and Methods

### 3.1. General Experimental Procedures

Column chromatography (CC) was performed with silica gel (200–300, 300–400 mesh, Qingdao, China). Thin-layer chromatography (TLC) was carried out with silica gel GF-254 plates (Qingdao, China). Ultraviolet (UV) spectra were recorded on a UV2550 UV/Vis spectrometer (SHIMADZU, Kyoto, Japan). The infrared (IR) spectra (KBr) were measured using a FTIR-8400S spectrophotometer (SHIMADZU, Kyoto, Japan). The optical rotations were determined in MeOH at 20 °C, using a PerkinElmer 341 digital polarimeter (Waltham, MA, USA). Circular dichroism (CD) spectra was carried out on a J-815 spectropolarimeter (JASCO, Kyoto, Japan). The MS data were determined on a LTQ-Obitrap XL (Thermo Scientific, Bremen, Germany) mass spectrometer for HRESIMS. The obtained 1D and 2D nuclear magnetic resonance (NMR) spectra were performed on a AVIII 600 spectrometer with TMS as the internal standard (Bruker Bispin Corporation, Fallanden, Switzerland).

### 3.2. Plant Material

The stems of the *K. interior* plants were collected from Fengqing City, Yunnan Province, China, in October 2015 and were identified by Prof. Ben-gang Zhang. A voucher specimen (NO. ID-KT-FQ201510) was deposited in the Resource and Conservation Research Center, Institute of Medicinal Plant Development, Beijing, China.

### 3.3. Extraction and Isolation

The dried and powdered stems (8 kg) of the *K. interior* plants were extracted three times with 90% EtOH. The extract was concentrated under a reduced pressure to dryness, which was then partitioned between EtOAc and H_2_O to provide the EtOAc-soluble fraction. The EtOAc fraction (243 g) was purified by CC on silica gel (200–300 mesh; petroleum ether/acetone gradient) to afford eight fractions, as follows: Fr_1_–Fr_8_. Fr_5_ was applied to silica gel CC with PE/EtOAc and was then separated by Sephadex LH-20 CC with CHCl_3_/MeOH (3:2 *v*/*v*) and preparative HPLC with MeOH/H_2_O, so as to yield compounds **1** (26 mg), **2** (77 mg), **3** (15 mg), **4** (20 mg), **5** (6 mg), and **6** (120 mg).

#### 3.3.1. Kadsutherin E (**1**)

White powder: [*α*${]}_{D}^{22}$ = −10 (*c* 0.07, MeOH). UV (MeOH, λ_max_, nm) (log ε): 221 (0.56). IR (KBr, υ_max_, cm^−1^): 3479 (-OH), 2951, 2840(CH), 1713 (C=O), 1504, 1450 (C-O). HR-ESIMS: *m*/*z* = 515.1691 [M + Na]^+^ (calculated for C_28_H_28_O_8_Na: 515.1690). ^1^H and ^13^C-NMR data (CD_3_OD) were shown in Table 1 and Table 2.

#### 3.3.2. Kadsutherin F (**2**)

White powder: [*α*${]}_{D}^{22}$ = 131.2 (*c* 0.08, MeOH); UV (MeOH, λ_max_, nm) (log *ε*): 221 (0.69); IR (KBr, υ_max_, cm^−1^): 3426 (-OH), 2956, 2928 (CH), 1669 (C=O), 1623 (C=O), and 1247 (C-O); and HR-ESIMS: *m*/*z* = 521.1791 [M + Na]^+^ (calculated for C_28_H_28_O_8_Na: 521.1792). ^1^H and ^13^C-NMR data (CD_3_OD) are shown in Table 1 and Table 2.

#### 3.3.3. Kadsutherin G (**3**)

White amorphous powder: [*α*${]}_{D}^{22}$ = −126.0 (*c* 0.05, MeOH); UV (MeOH, λ_max_, log *ε*): 221 (0.70) nm; IR (KBr, υ_max_, cm^−1^): 3411 (-OH), 2946, 2836 (CH), 1714 (C=O), 1648(C=O), 1265 (C-O); and HR-ESIMS: *m*/*z* = 543.1647 [M + Na]^+^ (calculated for C_29_H_28_O_9_Na: 543.1645). ^1^H and ^13^C-NMR data (CD_3_OD) are shown in Table 1 and Table 2.

#### 3.3.4. Kadsutherin H (**4**)

White amorphous powder: [*α*${]}_{D}^{22}$ = 47.5 (*c* 0.12, MeOH); UV (MeOH, λmax, nm) (log *ε*): 221 (0.54); IR (KBr, υ_max_, cm^−1^): 3434 (-OH), 2951 (CH), 1643(C=O), 1232 (C-O); and HR-ESIMS: *m*/*z* = 481.1478 [M + Na]^+^ (calculated for C_24_H_26_O_9_Na, 481.1476). ^1^H and ^13^C-NMR data (CD_3_OD) are shown in Table 1 and Table 2.

### 3.4. Anti-Platelet Aggregation Assay

The platelet aggregation assay was carried out according to the methodology that was reported in previous studies [17]. The blood was collected by catheterization of the abdominal aorta in rats (mean weight: 232.9 ± 4.8 g), anticoagulated with acid citrate dextrose (ACD) (9:1, *v*/*v*) and centrifuged for 15 min at 100× *g* at room temperature, so as to obtain platelet rich plasma (PRP). The platelet numbers were counted by a Coulter counter and were adjusted to 5.0 × 10^5^ platelets/μL. The platelet aggregation was measured at 37 °C using the turbidimetric method. The assays were performed at 37 °C in cuvettes using 300 μL of PRP under stirring, and the aggregation was triggered by the addition of adenosine diphosphate (ADP). All of the tested compounds (1mg/mL) were dissolved in 0.5% dimethyl sulfoxide (DMSO) [18] and were incubated with PRP for 2 min at 37 °C, and then, the platelet aggregation was triggered by adding ADP (10 μM). Aspirin was used as a positive control. The percentages of the inhibition were calculated as follows:

Inhibition (%) = [OD(control) − OD(compound)]/OD(control) × 100%.

The results were expressed as the mean ± SD and all of the anti-platelet aggregation data were statistical analyzed by SPSS (version 19.0, Chicago, IL, USA).

## 4. Conclusions

Four new lignans, named kadsutherin E–H (**1**–**4**), together with two known lignans (**5**–**6**), were isolated from the stems of the *K. interior* plants. The structures of those compounds were established on the basis of spectroscopic data. The anti-platelet effects of the six compounds were evaluated by suppressing the ADP-induced platelet aggregation in washed rat platelets. The results of the anti-platelet aggregation experiments indicated that compounds **1**–**6** could inhibit ADP-induced platelet aggregation at 100 μM. Kadsutherin F (**2**) showed the strongest anti-platelet aggregation activity with an inhibition of 49.47%.

## Figures and Tables

**Table 1:** ^1^H-NMR data of compounds **1**–**4** (CD_3_OD, *δ* in ppm, *J*/Hz, 600 MHz).

Position	1	2	3	4
4	6.60 (1H, s)	6.31 (1H, s)	6.49 (1H, s)	6.34 (1H, s)
6a	2.72 (1H, d, 13.8)	4.13 (1H, d, 10.8)	4.26 (1H, d, 10.2)	4.11 (1H, d, 10.2)
6b	2.77 (1H, dd, 13.8, 6.6)	-	-	-
7	2.20 (1H, overlap)	1.56 (1H, m)	1.66 (1H, m)	1.49 (1H, m)
8	2.20 (1H, overlap)	2.11 (1H, m)	2.18 (1H, m)	2.05 (1H, m)
9	5.92 (1H, d, 7.2)	5.61 (1H, d, 7.8)	5.72 (1H, d, 7.2)	5.86 (1H, d, 7.8)
11	6.49 (1H, s)	6.39 (1H, s)	6.48 (1H, s)	6.34 (1H, s)
17	1.11 (3H, d, 7.2)	1.02 (3H, d, 7.2)	1.08 (3H, d, 6.6)	0.92 (3H, d, 7.2)
18	1.15 (3H, d, 7.2)	1.04 (3H, d, 7.2)	1.17 (3H, d, 7.8)	1.02 (3H, d, 7.2)
19	5.97 (2H, d, 1.2)	5.99 (2H, s)	6.02 (2H, s)	6.00 (2H, s)
20	-	4.46, 5.57 (2H, ABq, 8.4)	4.40, 5.54 (2H, ABq, 8.4)	4.57, 5.62 (2H, ABq, 8.4)
1-OMe	-	3.85 (3H, s)	3.60 (3H, s)	3.89 (3H, s)
2-OMe	3.40 (3H, s)	3.69 (3H, s)	2.86 (3H, s)	3.76 (3H, s)
3-OMe	3.96 (3H, s)	-	-	-
1-OH	5,74 (1H, s)	-	-	-
6-OH	-	4.85 (1H, br s)	4.68 (1H, br s)	4.67 (1H, br s)
14-OH	5.26 (1H, s)	-	-	-
Acetoxy				
2′				1.79 (3H, s)
Angeloyl				
3′		5.87 (1H, m)		
4′		1.77 (3H, dd, 7.2, 1.2)		
5′		1.66 (3H, t, 4.8)		
Benzoyl				
3′,7′	7.43 (2H, dd,7.2, 1.2)		7.76 (2H, dd, 7.8, 1.2)	
4′,6′	7.30 (2H, dd, 7.2, 7.2)		7.40 (2H, dd, 7.8, 7.8)	
5′	7.48 (1H, dd, 7.2, 7.2)		7.57 (1H, dd, 7.8, 7.8)	

**Table 2:** ^13^C-NMR data of compounds **1**–**4** (CD_3_OD, *δ* in ppm, *J*/Hz, 150 MHz).

Position	1	2	3	4
1	138.2	168.3	169.5	169.8
2	134.2	134.8	136.0	136.2
3	151.2	185.5	186.6	187.0
4	107.0	131.8	134.6	156.8
5	133.8	155.3	131.6	131.5
6	38.3	80.8	81.8	78.9
7	34.8	38.7	44.0	40.4
8	42.2	42.3	39.8	43.3
9	83.5	79.2	82.1	82.2
10	117.8	130.1	122.0	122.2
11	101.1	100.2	101.7	101.3
12	147.9	150.6	152.0	156.8
13	135.2	129.8	131.3	130.0
14	134.4	145.3	146.5	156.8
15	147.6	120.4	156.4	146.6
16	117.3	57.9	59.2	59.0
17	18.5	18.2	19.6	19.4
18	14.3	9.11	10.4	9.1
19	100.2	102.0	103.4	103.2
20	-	83.7	85.1	84.2
1-OMe	-	60.6	61.4	61.7
2-OMe	55.2	59.4	59.4	60.7
3-OMe	59.3	-	-	-
1′	167.3	168.1	168.5	171.9
2′	129.7	136.8	130.9	20.8
3′	129.3	127.7	130.8	-
4′	127.8	14.6	130.0	-
5′	132.4	19.8	132.8	-
6′	127.8	-	130.0	-
7′	129.3	-	130.8	-

**Table 3:** Inhibitory effects of compounds on the aggregation of rat platelets induced by adenosine diphosphate (ADP) (100 μM, *n* = 9).

Compounds	Inhibition%
Kadsutherin E (**1**)	23.64 ± 1.12
Kadsutherin F (**2**)	49.47 ± 2.93
Kadsutherin G (**3**)	33.10 ± 2.67
Kadsutherin H (**4**)	21.75 ± 2.37
Acetoxyl oxokadsurane (**5**)	34.31 ± 0.73
Heteroclitin D (**6**)	11.77 ± 2.30
Aspirin	59.94 ± 2.44
